# 3D cell culture models: how to obtain and characterize the main models

**DOI:** 10.18699/vjgb-25-21

**Published:** 2025-04

**Authors:** М.M. Abdurakhmanova, A.A. Leonteva, N.S. Vasilieva, E.V. Kuligina, A.A. Nushtaeva

**Affiliations:** Institute of Chemical Biology and Fundamental Medicine of the Siberian Branch of the Russian Academy of Sciences, Novosibirsk, Russia; Sirius University of Science and Technology, Sirius Federal Territory, Krasnodar Region, Russia Institute of Chemical Biology and Fundamental Medicine of the Siberian Branch of the Russian Academy of Sciences, Novosibirsk, Russia; Sirius University of Science and Technology, Sirius Federal Territory, Krasnodar Region, Russia Institute of Chemical Biology and Fundamental Medicine of the Siberian Branch of the Russian Academy of Sciences, Novosibirsk, Russia; Institute of Chemical Biology and Fundamental Medicine of the Siberian Branch of the Russian Academy of Sciences, Novosibirsk, Russia; Sirius University of Science and Technology, Sirius Federal Territory, Krasnodar Region, Russia Institute of Chemical Biology and Fundamental Medicine of the Siberian Branch of the Russian Academy of Sciences, Novosibirsk, Russia

**Keywords:** cell aggregation, 3D cell cultures, spheroids, organoids, organ-on-a-chip, microtissue, 3D cell model culturing, агрегация клеток, 3D-культуры клеток, сфероиды, органоиды, орган-на-чипе, микроткань, культивирование клеточных 3D-моделей

## Abstract

For many years, the gold standard in the study of malignant tumors has been the in vitro culture of tumor cells, in vivo xenografts or genetically modified animal models. Meanwhile, three-dimensional cell models (3D cultures) have been added to the arsenal of modern biomedical research. 3D cultures reproduce tissue-specific features of tissue topology. This makes them relevant tissue models in terms of cell differentiation, metabolism and the development of drug resistance. Such models are already being used by many research groups for both basic and translational research, and may substantially reduce the number of animal studies, for example in the field of oncological research. In the current literature, 3D cultures are classified according to the technique of their formation (with or without a scaffold), cultivation conditions (static or dynamic), as well as their cellular organization and function. In terms of cellular organization, 3D cultures are divided into “spheroid models”, “organoids”, “organs-on-a-chip” and “microtissues”. Each of these models has its own unique features, which should be taken into account when using a particular model in an experiment. The simplest 3D cultures are spheroid models which are floating spherical cell aggregates. An organoid is a more complex 3D model, in which a self-organizing 3D structure is formed from stem cells (SCs) capable of self-renewal and differentiation within the model. Organ-on-a-chip models are chips of microfluidic systems that simulate dynamic physical and biological processes found in organs and tissues in vitro. By combining different cell types into a single structure, spheroids and organoids can act as a basis for the formation of a microtissue – a hybrid 3D model imitating a specific tissue phenotype and containing tissue-specific extracellular matrix (ECM) components. This review presents a brief history of 3D cell culture. It describes the main characteristics and perspectives of the use of “spheroid models”, “organoids”, “organ-on-a-chip” models and “microtissues” in immune oncology research of solid tumors.

## Abstract (ru)

В течение многих лет золотым стандартом в исследованиях злокачественных новообразований являлись культуры опухолевых клеток in vitro, ксенотрансплантаты in vivo или генетически модифицированные модели животных. К настоящему времени арсенал инструментов современных медико-биологических исследований пополнился трехмерными клеточными моделями (3D-культуры). 3D-культуры воспроизводят тканеспецифичные характеристики топологии ткани, что делает их релевантными тканевыми моделями с точки зрения клеточной дифференцировки, метаболизма и развития лекарственной устойчивости. Благодаря своему потен циалу такие модели уже применяются многими исследовательскими группами как для фундаментальных, так и для трансляционных исследований, и их использование позволяет значительно сократить количество экспериментов на животных, например, в области онкологии. В литературе 3D-культуры классифицируют по технике формирования (с каркасом/без каркаса), условиям культивирования (статические/динамические), а также по клеточной организации и функциям. По клеточной организации 3D-культуры разделяют на «сфероидные модели», «органоиды», «органы-на-чипе» и «микроткани». При этом каждая из моделей имеет свои характерные особенности, которые необходимо учитывать при использовании модели в эксперименте. Наиболее простые 3D-культуры – это «сфероидные модели», представляющие собой плавающие сферические агрегаты клеток. Более сложной 3D-моделью является «органоид» – самоорганизующаяся трехмерная структура, сформированная из стволовых клеток, способных к самообновлению и дифференцировке в составе модели. Микрофлюидные системы «орган-на-чипе» – это чипы, имитирующие in vitro основные физические и биологические процессы в органах и тканях в динамике. «Сфероиды» и «органоиды» за счет объединения различных типов клеток в единую структуру могут быть основой для формирования «микроткани» – гибридной 3D-модели, воспроизводя- щей специфический тканевый фенотип и содержащей тканеспецифичные компоненты внеклеточного матрикса. В данном обзоре представлена краткая история развития метода культивирования клеток in vitro в 3D-формате, описаны основные характеристики и перспективы применения «сфероидных моделей», «органоидов», «органов- на-чипе» и «микротканей» для исследований в области иммуноонкологии солидных опухолей.

## Introduction

In the middle of the 20th century, the basic principles of in vitro cultivation of plant and animal cells were formed and diploid human cell lines were created (Jedrzejczak-Silicka, 2017). In the late 20th and early 21st century, 3D cell culturing methods were developed to construct cell models that more accurately reproduce the microenvironment in which cells reside in body tissues (Edmondson et al., 2014). 3D tumor cell culture techniques have been actively developing in recent decades. Compared to 2D cultures, modern 3D cell models are as close as possible to animal models and in vivo primary tumors in terms of the following characteristics: the apical-basal polarity of cells within the 3D model; expression level of cell genes responsible for physiological functioning of cells; heterogeneity of cellular composition; ability to secrete extracellular matrix (ECM) proteins and growth factors; drug resistance of the model and etc.

Researchers classify 3D cell cultures according to their spatial structure (Maliszewska-Olejniczak et al., 2019) and distinguish “spheroidal models”, “organoids”, “organ-ona- chip” models and “microtissues”. In published works, the terms “spheroid”, “organoid” and “microtissue” may be mistakenly used as synonyms (Simian, Bissell, 2017). However, it should be kept in mind that all of the above models have different or only partially overlapping cell sources, construction protocols and applications and as such are not interchangeable. The reasons why the terms “spheroid model”, “organoid” and “microtissue” need to be separated are described in this review. The review also presents a brief history of the development of in vitro 3D cell culturing methods with a focus on the key features of 3D cellular models, which will allow researchers to determine the most physiologically relevant model for cancer immunology studies of solid tumors.

## Preservation of tissue-specific characterization of cells in vitro

The first attempts to obtain a 3D cell model were made in 1956: Aron Arthur Moscona obtained 3D structures in the form of cell aggregates (Moscona, 1956). Moscona was the first to show that dissociated cells of different histological origin, when cultured together, are able to aggregate with each other and form a three-dimensional structure.

Radiobiologists Robert Sutherland et al. first introduced the term “spheroid” for the structures described by Aron Moscona. Sutherland and colleagues obtained multicellular spheroids from Chinese hamster lung cells (line V79). The structure of the resulting spherical cell aggregates resembled the nodules observed in animal and human carcinomas. The growth curve of cell aggregates in vitro was similar to the growth curve of grafts in mice. Morphological analysis of the obtained structures showed that spheroids have an outer zone containing many dividing cells, an intermediate zone, which is poorly saturated with oxygen and nutrients and contains a small number of cells in the state of mitosis, and a zone of necrotized cells. Based on the results obtained, the authors concluded that the multicellular spheroids obtained during the experiment can be used as an in vitro model to assess tumor growth (Sutherland, 1988).

The term “organoid” began to be used in the literature in the 1950s, but, at that time, the structures denoted by the term had nothing to do with “3D cell cultures”. For example, William Duryee and Josephine Doherty, in their 1954 study “Nuclear and Cytoplasmic Organoids in the Living Cell”, used the term “organoid” to refer to intracellular structures, namely cell organelles (Duryee, Doherty, 1954). The term “organoid” was also used to refer to tumors or abnormal cellular growths as a synonym for “teratoma” (Wolter, 1967). The development of methods for culturing organoids as 3D cellular structures dates back to 1975. James G. Reinwald and Howard Green described the first 3D model that contained normal human keratinocytes and mouse fibroblasts of the 3T3 line. In the stratified epidermis, cell division was restricted to the basal layer of growing clones, while the superficial layers consisted of terminally differentiating keratinocytes that gradually formed the keratinizing layer. Further culturing of these structures yielded “epidermal sheets” grown from small numbers of primary keratinocytes (Rheinwatd and Green, 1975). Although the term “organoid” was not used in this study, Rheinwatd and Green were the first to reconstruct a 3D tissue structure in vitro, and since 1980, the term “organoid” has appeared in studies on 3D cultures

In addition, in the 1980s, the work of a group led by Mina Jahan Bissel demonstrated the important role of ECM in tumor development. Primary culture mouse mammary gland cells were cultured on a substrate of basal membrane (BM) proteins derived from Engelbreth-Holm-Swarm (EHS) mouse sarcoma. It was shown that in this conditions mammary cells formed ducts and lumen resembling secretory alveoli, and β-casein expression was detected in 90 % of the cells (Li et al., 1987). This study stimulated the development of methods to create 3D models with the consideration of the ECM. The combination of the words “3D cell culture models” was first used by Mary Helen Barcellos-Hoff et al. (Barcellos-Hoff et al., 1989) and Ole Petersen and colleagues (Petersen et al., 1992) when analyzing mammary gland cells on EHS BM substrate. Using this human mammary gland model, the group led by Barcellos-Hoff investigated alveolar morphogenesis, and the group led by Petersen was able to describe the growth pattern and differentiation of normal and malignant epithelial cells.

Until 2005, the term “organoid” was used to refer to small organ fragments consisting mainly of epithelial cells separated mechanically and/or enzymatically from stromal tissue and grown in various gels (Fata et al., 2007). However, in the last decade, the term has often been used to refer to a wider variety of 3D structures (Nikonorova et al., 2023). In 2012, The Gastrointestinal Stem Cell Consortium approved the following nomenclature for cell models of the large and small intestine: “organoid” – a 3D culture consisting of several cell types, such as cells of epithelial and mesenchymal origin; “spheroid” – a spherical 3D culture containing cells of only one cell type (Guryanov, 2016).

To clarify the nomenclature of cellular models for other tissues, the European Molecular Biology Organization organized the “Organoids” meeting in October 2016, where it was decided to apply the term “organoid” to a range of different structures, depending on the organ system (Simian, Bissell, 2017). For example, in the field of mammary gland biology, an “organoid” is a primary explant of epithelial ducts placed in ECM gels. Conversely, in intestine biology research, “organoids” may include clonal derivatives of primary epithelial stem cells (SCs) grown without mesenchyme or epithelial-mesenchymal cultures derived from embryonic stem cells (ESCs) or induced pluripotent stem cells (iPSCs) (Shamir, Ewald, 2014).

**Fig. 1.**
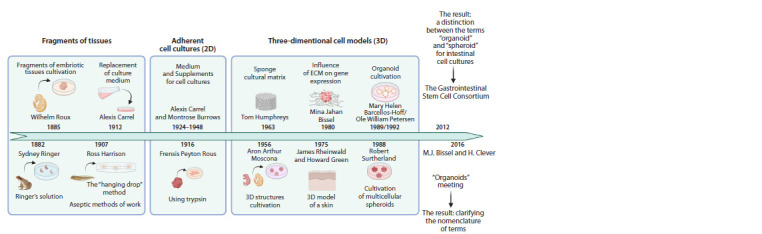
Chronology of key developments in cell culturing: from tissue fragments to 2D and 3D cell models.

Thus, the methods of tissue fragment cultivation developed and described in the 19th and 20th centuries laid the foundation for the development of cell culture technology outside the body. The formulated principles of cell cultivation allowed to make important discoveries in the field of regenerative medicine, transplantology, biotechnology and biopharmaceutics (Simian, Bissell, 2017) (Fig. 1).

## The specific features of 3D tumor cell cultures: “spheroid model”, “organoid”, “organ-on-a-chip” and “microtissue”

With the development of 3D culturing approaches, terms such as “aggregates”, “spheroids”, “sphere”, “tumorsphere”, “oncosphere”, “organoid” or “organotypic spheroid” appear. They are often mistakenly used as synonyms. However, these models differ in the composition of the medium used, the cell culture surface, the cell density, the time required for formation, and the types of cells used (Rodrigues et al., 2024). That said, the ambiguity of the terminology can lead to confusion about the specific model used in a given study (Nikonorova et al., 2023). For example, Seyed Ali Karimifard et al. use the terms “organoid” and “mammosphere” in reference to a 3D cellular structure from MCF-7 breast adenocarcinoma tumor cells (Karimifard et al., 2024). According to the nomenclature of cellular 3D structures, “organoid” and “mammosphere” refer to different 3D models (Ponti et al., 2005; Gilazieva et al., 2020). The authors of this study refer to the publication by Sahar Moradi-Mehr et al. who describe engineered “mammospheres” as an organoid model (Moradi-Mehr et al., 2023). However, the authors of this work do not describe the model they obtained as an “organoid”, but use the terms “3D MCF-7 cell culture” or “mammosphere”.

**Fig. 2.**
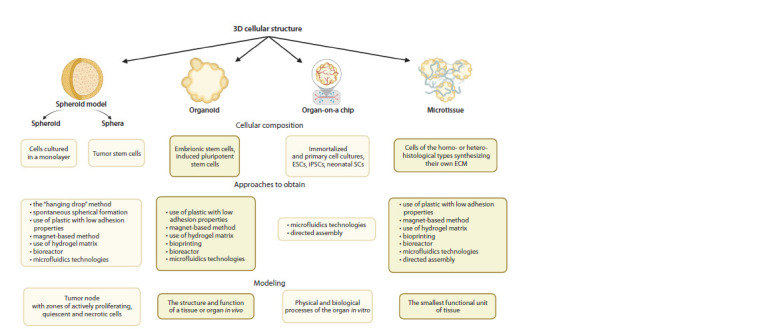
Methods of obtaining and characteristic features of 3D cellular structures: “spheroid model” , “organoid” , “organ-on-a-chip” and “microtissue” .

We assume that the confusion in terminology is related to the novelty and speed of development of the field of 3D cell culture, as well as the desire to follow scientific trends. The importance of using appropriate terminological nomenclature was also discussed in a scientific review by V.G. Nikonorova et al. (Nikonorova et al., 2023). Despite numerous attempts to introduce nomenclature, the use of terminology is rather inconsistent among researchers; therefore, it is necessary to introduce nomenclature of cell models in the scientific community, including among Russian researchers (Kang et al., 2021; Pașca et al., 2022) (Fig. 2).

## Spheroid models

Among spheroidal models, “spheres” and “spheroids” are the most common (Maliszewska-Olejniczak et al., 2019). “Spheres” include tumorospheres and tissue tumor “spheres”. Tumorospheres are described as tumor cells forming 3D clusters of cell suspension growing under non-adhesive conditions. Tumor stem cells (CSC), which are associated with tumor initiation, have the potential for self-renewal and proliferation, as well as the ability to form 3D structures when cultured in vitro (Weiswald et al., 2015). Since sphere-forming cells are SCs, they are able to differentiate into all non-stem cell subpopulations present in the original cell culture, and thus, a tumorosphere is a mixture of CSC and differentiated cells (Maliszewska- Olejniczak et al., 2019). By contrast, tissue tumor “spheres” are derived from a patient’s tumor tissue sample. The tissue sample is dissociated, allowing tumor cells to migrate from fragments as clusters of cells and/or individual cells to form dense, compact clusters or aggregates of cells. However, this spheroid model is limited to the study of the CSC region, as it cannot reproduce the multiplicity of other cell types in a tumor, and is also poorly reproducible as some CSC remain undifferentiated (Valent et al., 2012).

“Spheroids” are aggregates of cells of a spherical shape formed in a suspension of single cells of homo- or heterogeneous cell type. The formation of such a model occurs due to homotypic intercellular adhesion, complemented by the lack of cell adhesion to the plastic of the culture vial (Sakalem et al., 2021). Such a 3D model can be formed from cells of the same lineage as well as from cells of different lineages cultured together, and allows us to assess the ability of cells to spontaneously self-organize, synthesize ECM proteins and form a specific microenvironment (Verjans et al., 2018). The spheroid resembles a non-vascularized tumor nodule – it mimics the central zone of hypoxia, the inner zone of quiescent cells and the outer zone of actively proliferating cells and is convenient as a model in the study of malignant neoplasms.

The main application area of spheroid models: in biological research as an in vitro tumor model, for drug testing, as a basis for tissue engineering (Daly et al., 2021; Hsu et al., 2021; Corgnac et al., 2022; George et al., 2022; Nushtaeva et al., 2022; Vasileva et al., 2022).

## Advantages and disadvantages of the spheroidal model

One of the advantages of spheroid models is that they do not require an exogenous ECM (Nushtaeva et al., 2022). Such models reproduce the biochemical reactions of the original parental tumor (George et al., 2022) and intercellular interactions (Corgnac et al., 2022). In addition, spheroid models can be used as building blocks for organ-on-a-chip models and microtissues (Corgnac et al., 2022).

However, it is important to consider that depending on the method of derivation, the duration of cultivation and the size of the spheroid, the necrotic area may also increase, limiting researchers, for example in studies related to drug testing (Verjans et al., 2018). Also, not all cell lines are able to form spheroid models (Ivascu, Kubbies, 2007) and there is limited availability of cell lines derived from normal or minimally transformed tissues (Gunti et al., 2021; Han et al., 2021). In addition, a detailed selection of growth factors is required for the formation and maintenance of the spheroid model.

## Prospects for the application of “spheroid models” in immunologic and cancer research

Over the last decade, immunotherapy has become a promising tool in oncotherapy (Bandara et al., 2024). Despite this, the efficacy of immunotherapy often depends on tumor histogenesis and patient characteristics. This suggests the need for improved preclinical screening models that more accurately reproduce tumor biology in vivo

Spheroid models can be grown either from tumor cells alone or co-cultured with different cell types such as fibroblasts, endothelial cells, and immune cells to mimic crosstalk between different cellular compartments of patients’ tumors (Abdurakhmanova et al., 2022; Heinrich et al., 2024). Although spheroids lack the vasculature and cellular heterogeneity of the primary tumor, their gene expression profiles and necrotic core formation make them similar to patients’ tumors (Heinrich et al., 2024). It is currently the most used model to evaluate immunotherapeutic strategies due to its relatively low cost and high reproducibility (Boucherit et al., 2020).

Spheroid models can be used to test immunotherapy approaches, particularly to assess the efficacy of therapeutic antibodies and carry out drug screening to enhance immune cell infiltration and antitumor effects against solid tumors. For example, in the study by Melanie Grotz and colleagues, a heterotypic spheroid model of breast cancer was used to evaluate the effect of a high-affinity ligand of fibroblast activation protein on naive T-cell behavior (Grotz et al., 2024). This study showed that targeting the fibroblast activation protein is relevant for immunotherapy and effective activation of T cells in the tumor microenvironment. Spheroid models can also be used to test the efficacy of the chimeric antigen receptor (CAR) therapy approach. Veronica Bandara et al. tested their CAR-T cells targeting the non-functional purinergic receptor P2X7 and found that this approach enhanced the anti-tumor response in a spheroid model of ovarian cancer (Bandara et al., 2024). Spheroid models can also be used to investigate the role and functions of nanoscale biomolecules. In the study by Lilita Sadovska and colleagues, a 3D cellular model was developed to evaluate the effects of extracellular vesicles (EVs) in prostate cancer on human immune cells (Sadovska et al., 2018). The study showed that the majority of EVs remain bound on the surface of B cells, while a part of EVs penetrate into T cells via macropinocytosis.

In addition to generating spheroids derived from tumor cells, another approach is to develop spheroids derived from immune cells. Macrophages form spheroids and can remain viable in 3D culture for at least 16 days (Burchett et al., 2024). Y. Tanaka et al. were able to demonstrate that macrophages tend to polarize towards the anti-tumor M1 phenotype, opposing its pro-tumor M2 phenotype in the spheroid state (Tanaka et al., 2018).

However, in order to accurately mimic tumor composition and investigate the functional properties of immune cells, it is necessary to improve existing spheroid models. For example, by introducing new cell types into the spheroid in a quantitatively accurate manner. In addition, the cell ratios in the model must match what the tumor exhibits. This requires extensive study of the cellular composition of the tumor before creating the model. The most comprehensive heterotypic spheroid model was created in a study by Marcel Heinrich et al. (Heinrich et al., 2024). The authors of this study determined the number and ratio of glioblastoma tumor cells, microglia, and astrocytes to recreate a realistic brain tumor model. The inclusion of both astrocytes and microglia in the heterotypic model significantly increased the growth of the model, and demonstrated that astrocytes play a crucial role in glioblastoma cell invasion. In addition, astrocytes and microglia contribute to a dense physical barrier that protects the tumor model from infiltration by macromolecules or immune cells.

## Organoids

A significant part of 3D cell cultures is called “organoids” because, under the conditions of mimicking the 3D environment of an organism in vitro, cells can spontaneously selforganize, forming complex histological structures similar to the structures in the organs from which they originated. For example, mammary gland cells cultured in 3D are able to form structures similar to branched ducts (Lee et al., 2007). Currently, the term “organoid” refers to an artificial 3D structure derived from SCs and composed of organspecific cells capable of self-organization and reflecting the structure and function of an organ in vivo. Such a model can be derived from ESCs, iPSCs or neonatal SCs (Sakalem et al., 2021; George et al., 2022) and provides relevant insights into tissue functionality and differentiation. Typically, “organoids” are composed of different cell types originating from different germ sheets and tend to have a higher order of self-organization compared to spheroids (Nikonorova et al., 2023).

When describing “organoids”, the term “assembloids” is also used – uniting organoids formed from cells of different organs or different regions of an organ (Eke et al., 2022). Such a model should mimic the morphofunctional units of the corresponding tissues in vivo.

The main application area of organoids: biomedical research, drug testing, tissue engineering and transplantation therapy (Kassis et al., 2019; Hofer, Lutolf, 2021; Mesci et al., 2022; Miao et al., 2022).

## Advantages and disadvantages of organoids

By altering the cell isolation procedure and varying the combination of growth factors during culturing, researchers can create organoids composed of both normal and transformed cells (Ivascu, Kubbies, 2007; Daly et al., 2021; Hsu et al., 2021; Corgnac et al., 2022), which is a powerful tool in antitumor drug screening studies. Cellular models of “organoids” can be cultured for long periods of time, genetically modified and cryopreserved, preserving their phenotypic and functional characteristics. However, it should be taken into account that the formation of a complex structure in the “organoid” model usually takes two to three months depending on the tissue type and requires a certain set of growth factors (Gunti et al., 2021).

## Prospects for the application of “organoids” in immunologic and cancer research

The use of patient-derived organoids in personalized cancer immunotherapy has shown great potential. Such organoids retain the genetic and functional characteristics of the original tumors, allowing immunotherapeutic strategies to be tailored to each patient’s unique cancer profile (Noorintan et al., 2024).

A study by S.D. Forsythe et al. used personalized organoid models to preclinically investigate the use of immunotherapy in the treatment of appendix cancer (Forsythe et al., 2021). Patient tumor organoids were generated using unsorted tumor cells with and without enrichment of patient immune cells derived from peripheral blood, the spleen, or lymph nodes for therapy with PD-1 (programmed cell death protein 1) inhibitors and T-cell activators. The authors demonstrated cytotoxic efficacy in a subset of immuneenhanced appendix cancer organoids from both low and high malignancy primary tumors. This study demonstrates the potential of immunotherapy for appendix cancer and the utility of immunocompetent organoids in selecting patients for clinical trials in rare cancers.

Incorporation of 3D models to predict clinical responses to screening drugs turned out to be more effective than use of traditional adherent cultures, as 3D models reproduce the features of the primary tumor to a greater extent. Z. Zhou et al. developed a standardized protocol to establish a tumororganoid- T-cell system with breast tumor organoids and primary tumor-specific CD8+ T cells. This system facilitates high-throughput drug screening using mouse mammary tumor organoids and also allows for more accurate prediction of therapeutic responses to anticancer drugs using personalized organoids (Zhou et al., 2021). The authors showed that current epigenetic inhibitors enhance antigen presentation mediated by major histocompatibility complex class I (MHC I) on breast tumor cells. Furthermore, treatment with the histone deacetylase inhibitor BML-210 significantly sensitized breast tumor cells to the PD-1 inhibitor.

Developing co-culture systems for primary tumor epithelium that include additional cellular components without artificial addition is challenging. J.T. Neal et al. successfully created organoids derived from patient tumor epithelium that retain their own immune cells, reflecting the diversity of the tumor microenvironment (Neal et al., 2018). Populations of infiltrating CD3+ T cells expressing PD-1, cytotoxic T cells, T helper cells, T cells, B cells, NK cells and varying numbers of macrophages were observed in the personalized organoids. This method holds great promise for modeling personalized immunotherapy in vitro by organoids that retain their immune structure.

T.E. Schnalzger et al. developed organoids from patientderived colon cells to study the cytotoxicity of CAR-NK cells targeting the EpCAM (cell adhesion molecule) antigen (Schnalzger et al., 2019). CAR-NK-EpCAM effectively lysed tumor cells on the first day of co-culture. The authors claim that the organoids they obtained represent a sensitive, personalized in vitro platform for evaluating the efficacy of CAR-based immunotherapy.

However, no matter how sophisticated organoid models are, they do not provide a physiological representation of tissue organization in vivo. In these models, there is no vascular system, and consequently, the diffusion of drugs, cellular products and their penetration inside the organoid is limited.

## Organ-on-a-chip

Organ-on-a-chip technology has revolutionized biomedical research by providing advanced platforms for in vitro modeling of complex organ systems. “Organ-on-a-chip” is a technology for culturing cells in a fluid flow to mimic an artificial organ or their system, allowing the structural and functional characteristics of organs and their interactions to be reproduced. This technology is applicable to the study of disease mechanisms, responses of body systems to therapeutic agents and their toxicity profiles (Doost, Srivastava, 2024).

The organ-on-a-chip model is a small microfluidic device in the form of chips made of biocompatible materials that, through a network of microchambers, microchannels, and laminar flow, allow cells to be cultured under conditions similar to in vivo environments (Doost, Srivastava, 2024). Such a model can be derived from ESCs, iPSCs or neonatal SCs, as well as immortalized and primary cell cultures (Singh et al., 2022). In addition, microfluidic technologies can be combined with a “spheroid model” and/or “organoids” to form a hybrid model (Wei et al., 2023).

The main application area of organ-on-a-chip: biomedical research, drug testing, tissue engineering (Azizgolshani et al., 2021; Lohasz et al., 2021).

Advantages and disadvantages of the organ-on-a-chip model

“Organ-on-a-chip” allows full control of microfluidic systems and regulation of cellular processes in a study, mimicking dynamic human physiological processes such as respiration, peristalsis, and blood flow (Alver et al., 2024).

One of the limitations of organ-on-chip technology is the need for a material that does not affect the components of the cellular microenvironment and maintains a stable fluidic connection. Since the volume of laminar fluid is small, surface effects dominate over volume effects. In addition, laminar flow is present at the intersection of multiple fluids, and consequently the fluids may not mix properly (Danku et al., 2022).

Prospects for organ-on-a-chip application in immunologic and cancer research

Blood and lymphatic vessels play an important role in immunologic processes, moving immune cells between organs, tissues, and the lymphatic system. Microfluidic chip technology can replicate key complex and dynamic tumor characteristics such as vascularization and extravasation, improving preclinical models in the development of cancer immunotherapy (Doost, Srivastava, 2024). Most organon- a-chip models contain parallel channels to incorporate tumor cells into hydrogels and immune cells embedded in the hydrogel or perfused from the side channel. The specific choice of microfluidic model design is usually determined by the purpose of investigation, as throughput, dynamic characteristics (e. g., flow), and molecular sensing capabilities vary widely between models (Chernyavska et al., 2023). Shabnam Jeibouei et al. used spheroids formed from breast cancer cells in a microfluidic chip to assess patient tumor heterogeneity and analyze migration and invasive potential (Jeibouei et al., 2024). The authors found that increased expression levels of HER2 and the macrophage marker M2a as well as the stiffness of VSMC proteins are important factors affecting tumor cell migration and invasion. M. Nguyen and colleagues reconstructed a heterotypic HER2+ breast tumor model to evaluate the effect of monoclonal antibodies. The authors cultured tumor cells, endothelial cells, blood mononuclear cells, and tumor-associated fibroblasts in a multichamber chip. This model allowed testing of monoclonal antibodies in a complex 3D system that allows perfusion of soluble molecules given the heterogeneity of the tumor (Nguyen M. et al., 2018).

Unlike adaptive immune cells, innate immune cells do not need MHC for their activation. The complexity increases significantly when adaptive immune cells have to be used in an experiment, given MHC molecules, in the presence of other MHC-mismatched cell types (Magenau et al., 2016). It is therefore crucial to develop immunocompetent organon- a-chip models to help us better understand how immune cells interact with organs in health and disease. Research by Irina Veith and colleagues created personalized organ-on-achip models of lung cancer with their autologous primary tumor, stromal, and immune cells isolated from tumor samples and measured the response to anti-PD-1 treatment (Veith et al., 2024). The microfluidic model was able to reproduce stroma-dependent mechanisms of resistance to immunotherapy, and integration of autologous immunosuppressive tumor-associated fibroblasts into the model impaired the response to anti-PD-1 therapy.

Although organ-on-a-chip models can reproduce most characteristics of individual organs and physiological flow conditions, it is unable to capture dynamic interactions between multiple organs (Kumar et al., 2024). In addition, an organ-on-a-chip still does not include all organ-specific cells and requires further refinement of the model, for example via integration of organoids into the model. Tengku Maulana created a model for infusion, recruitment and infiltration of CAR-T cells into solid tumors by integrating organon- a-chip approaches and patient-derived organoids. The model was used to investigate different treatment regimens with dasatinib as a pharmacologic safety switch to control CAR-T cells during therapy. The approach allowed in vitro evaluation of safety and efficacy in a patient-specific manner (Maulana et al., 2024).

## Microtissue

A “microtissue” is a hybrid cellular 3D model that has a tissue-specific phenotype and contains tissue-specific ECM components. “Microtissues” are formed when cells in a suspension aggregate with each other and/or bind to the surrounding ECM and compactify, increasing the density of the 3D structure (Eyckmans, Chen, 2017). It is possible to form a “microtissue” by obtaining a model of “spheroids” or “organoids” from both a single cell type and histologically different cell types (Eke et al., 2022), as well as by integrating into an organ-on-a-chip model. In this approach, “microtissues” can be spherical multicellular aggregates designed to replicate the smallest functional unit of a tissue or organ. During self-organization, cells synthesize their own ECM, re-establish cellular contacts, and thus reproduce tissue-specific functions and integrated cellular responses to environmental stimuli. Although the microtissue forms an environment that allows certain cell types to mimic their native in vivo behavior as closely as possible, many tissues in the body experience significant mechanical loading that alters matrix structure and cell function, which is difficult to reproduce in a 3D model (Eyckmans, Chen, 2017).

The main application area of microtissues: biomedical research, drug testing, tissue engineering and transplantation therapy (Wang Y. et al., 2020; Zhang et al., 2022).

Advantages and disadvantages of microtissue

Microtissues allow recreating complex native tissue architecture in vivo, including simulation of vascular network, cell-cell and cell-ECM interactions (Eke et al., 2022). Pathological processes are being modeled using microtissue for personalized screening and drug development. However, the low assembly speed for macroscale tissue simulation, building a scenario of cellular evolution in 3D dimension leading to the emergence of function rather than the formation of the final functional structure should be considered. In addition, the sources of initial cells can affect model fidelity and reproducibility (Eke et al., 2022; Schot et al., 2023; Wang O. et al., 2023).

Prospects for the application of “microtissues” in immunologic and cancer research

A microtissue is an in vitro biomimetic model formed from spheroids and/or organoids as biological building blocks for tissue and organ development, both through simple 3D culturing approaches and innovative engineering systems (Burdis et al., 2022). The advantage of a microtissue model is that the tissue organization can be fully engineered and the assembly of the model can be adjusted chemically or mechanically to obtain the desired tissue structure.

Claudia Martins and colleagues developed a spheroidbased heterotypic glioblastoma microtissue model to evaluate the effect of nanodrugs (Martins et al., 2023). The resulting model mimicked tumor organization, extracellular matrix production, and exhibited a cytokine signature. Macrophages within the microtissue were polarized into an M1/M2 phenotype consistent with docetaxel nanotherapy. In the study by Kazuaki Ninomiya and Tatsuhiko Taniuchi, a bio-3D printer with spheroid stacking on Kensan (microneedle matrix) was used and a microtissue was assembled by precisely stacking spheroids from normal and cancer cells. The resulting model allowed to non-invasively observe the dynamic invasion behavior of cancer cells for the first time (Ninomiya, Taniuchi, 2024). Inya Waldhauer et al. developed heterotypic 3D microtissue models to study the activity of novel IL-2-based anti-tumor immunotherapeutic drugs (Waldhauer et al., 2013). The resulting tumor cell/ fibroblast/lymphocyte-based microtissue model allows us to control the penetration of antibodies and their targeting of tumor and stroma components, to study the interaction of tumor cells with immune cells in a system that more closely resembles the tumor microenvironment in vivo. Using bioprinting and microfluidic emulsification systems, Gyusik Hong and colleagues obtained a microtissue spheroid model with a lobular structure and realization of liver functions (Hong et al., 2021). Structured microtissue spheroids with pronounced vascularization showed improved albumin and urea secretion

Thus, the use of the microtissue approach involves the combination of already existing 3D models to enhance the reproduction of realistic tissue features in the field of tumor immunology, and remains a promising model in the development of immunotherapy strategies.

## Cell culturing in 3D models

**Fig. 3.**
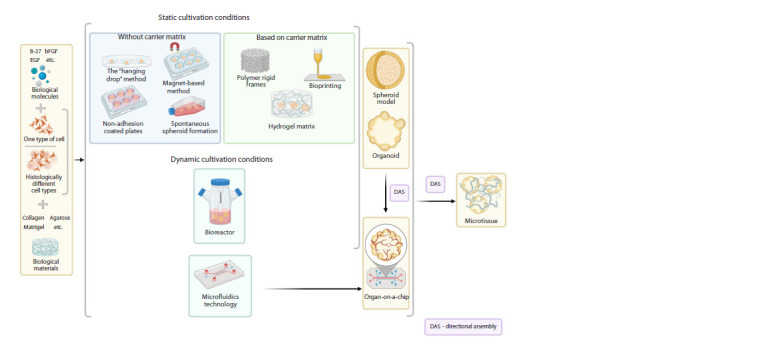
Methods of obtaining 3D structures.

Cultivation conditions in 3D systems should provide cells with all physical and chemical conditions necessary to mimic the in vivo environment. At present, there are many methods for culturing cells as part of 3D structures (Fig. 3). The following criteria should be considered when selecting a method for obtaining a 3D cell structure:

1) cell composition: a mono- (Troitskaya et al., 2021) or heterogeneous cell model (Arora et al., 2022; Nushtaeva et al., 2022); 2) method of 3D model formation: using special carrier matrices (Sulaiman et al., 2020) or without their use (Nushtaeva et al., 2022); 3) cultivation conditions: static (Arora et al., 2022) or dynamic (Coluccio et al., 2019).

Some advantages and disadvantages of methods for obtaining basic 3D models are summarized in the Table.

**Table 1.**
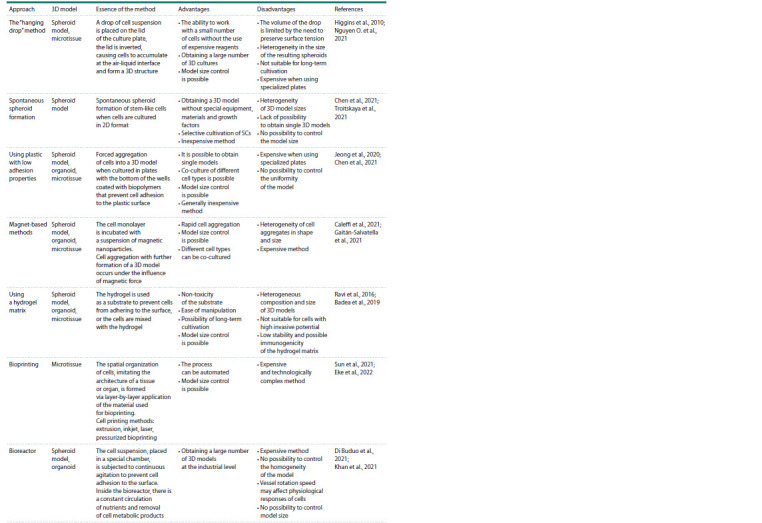
Advantages and disadvantages of methods of cultivation of basic 3D models

**Table 1.end:**
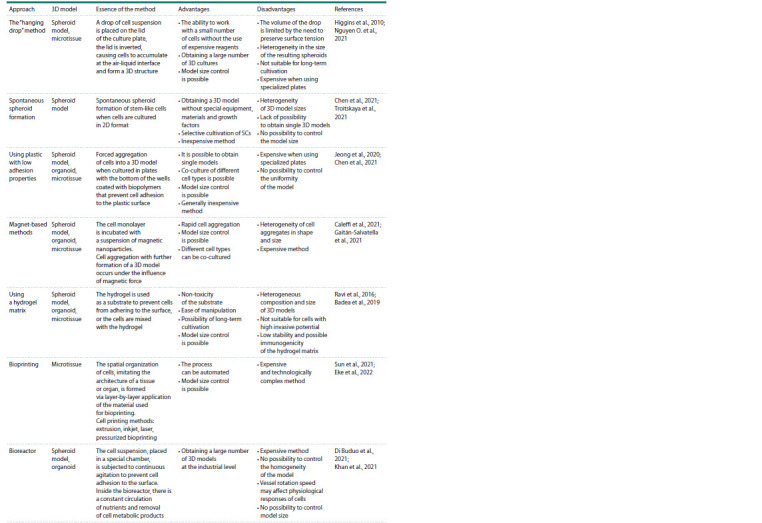
Table 1.end

## Conclusion

Compared to cells in adherent cultures, cells in 3D structures simulate intercellular interactions organized in space and cellular heterogeneity, which together more fully reflect tissue organization in vivo (Eke et al., 2022). This review discusses the nuances of terminology in 3D cell modeling, the main approaches to obtaining models, and the prospects for their use in biomedical research.

Three-dimensional “spheroid models” and “organoids” provide an opportunity to approximate the architecture and functionality of the tissue from which they originate. However, despite the advantages of these models to account for part of the microenvironment, such as stromal and immune cells, they still lack the environmental dynamics inherent to in vivo conditions. Organ-on-a-chip microfluidic technologies in the field of oncology combine the advantages of 3D culture in a controlled and dynamic environment. In addition, “spheroids” and “organoids” act as building blocks and form a “microtissue” that recreates the complexities of native tissue architecture in vivo (Eke et al., 2022).

Three-dimensional cellular models are an informative tool for investigating mechanisms of disease development and progression, as well as identifying novel biomarkers, since they are as close as possible to the primary tumor at the cellular and molecular genetic level. In addition, such models are a relevant preclinical in vitro platform for drug development and realization of the potential of personalized medicine

## Conflict of interest

The authors declare no conflict of interest.
